# Cell Programming for Cardiovascular Disease Modeling and Therapy

**DOI:** 10.3390/ijms24097877

**Published:** 2023-04-26

**Authors:** Robert David

**Affiliations:** 1 Department of Cardiac Surgery, Rostock University Medical Center, 18057 Rostock, Germany; robert.david@uni-rostock.de; Tel.: +49-381-4988973; 2 Department of Life, Light, and Matter of the Interdisciplinary Faculty, Rostock University, 18059 Rostock, Germany

Cardiovascular diseases have a high mortality due to a very limited regenerative potential of lost cardiomyocytes and therefore are one of the leading causes of death in developed countries. To increase the limited treatment options, new approaches to personalized regenerative therapy and drug development are therefore of great importance. In recent years, the targeted forward programming of induced pluripotent stem cells (iPSCs) and the direct reprogramming of somatic cells and adult stem cells have created entirely new opportunities to circumvent the problems often encountered in regenerative medicine by using autologous cells as a cellular source of therapy. This has greatly benefited from research efforts to identify and optimize master–regulator combinations to redefine cell fates. Several research groups have shown a direct conversion of somatic cells into cardiovascular cells, avoiding a pluripotent intermediate state. In addition, in vitro test systems based on organoid cultures obtained from cardiovascular cells that are programmed to be patient-specific are being further developed for personalized drug testing in precision medicine and in vitro disease modelling to further understand the underlying pathophysiological mechanisms. Against this background, the present Special Issue covered topics from understanding the basic science of somatic and stem cell reprogramming to their applications in cardiovascular regeneration and disease treatment, as well as approaches to modeling diseases in vitro. The new Special Issue titled “Cell Programming for Cardiovascular Disease Modeling and Therapy” of the *International Journal of Molecular Sciences* includes a total of nine contributions, with four original articles and five reviews, providing new information about cardiovascular cell programming, disease modeling and cell based therapy, provided by scientists that are active in the field.

State-of-the-art and new approaches toward direct cardiac reprogramming were extensively reviewed by López-Muneta et al. [1] starting with the initial combination of three transcription factors, GATA4, MEF2C and TBX5 (GMT cocktail), used for the reprogramming into induced cardiomyocytes in vitro using mouse fibroblasts. The article then discusses optimized programming factor combinations including microRNAs or small molecules relative to the original GMT cocktail as well as the programming of other cell types such as endothelial and smooth muscle cells to form new blood vessels. Moreover, direct cardiac reprogramming as opposed to in vitro programming has emerged as a novel therapeutic approach to treat and regenerate injured hearts via the direct conversion of fibroblasts into cardiac cells. With this aim, several studies have centered on the direct reprogramming of fibroblasts into induced cardiac progenitor cells (iCPCs) that are able to give rise to all myocardial cell lineages. These multipotent and highly expandable mouse iCPCs can be generated, suggesting that clinically relevant amounts of these cells could be created. In addition to the still outstanding generation of human iCPCs, it is necessary to determine the appropriate stage of maturity for a cell therapy product. Beyond that, poor retention, survival rate, and the implantation of the transplanted cells in the heart tissue are the biggest hurdles in regenerative medicine. To address these concerns, research is being conducted on alternative approaches, such as in vivo reprogramming, which have fewer barriers to their translation into the clinic. Since this method has produced better results in terms of efficiency and iCM maturity in mouse models, the cardiac environment is thought to be conducive to this process. Contributing to this approach, safer delivery systems such as Sendai virus, adenovirus, chemical cocktails or nanoparticles have been the subject of research in recent years.

With a different focus, Jiang et al. reviewed cardiac reprogramming-based progress as well as existing obstacles for generating CMs such as low efficiency, functional maturity and safety issues for clinical translation [2]. Importantly, this article also addresses tissue engineering approaches, the importance of thoroughly understanding signaling pathways underlying cardiac development, and epigenetic mechanisms as crucial prerequisites to establish methods for cell-reprogramming based heart regeneration.

Raziyeva et al. reviewed the discrepancy between initially promising results regarding the improvement of cardiac function post-myocardial infarction after stem cell transplantation and subsequent disappointing findings with respect to insufficient cell survival and low engraftment at the inflammatory and hostile environment at the site of infarction, which strongly limits the existing regenerative potential of transplanted cells [3]. Against this background, state-of-the-art knowledge related to utilizing preconditioned stem cells for myocardial infarction treatment, with a focus on hypoxic preconditioning, growth factor and drug application as well as the administration of biological agents is presented. Furthermore, genetic stem cell engineering, including programming factor and microRNA overexpression as a basis for cell optimization to improve their efficiency post MI, is discussed.

In their current review article, Galow et al. introduces an additional aspect: the genetic engineering of porcine cells and their future applications in xenogeneic heart regeneration as an alternative to the use of human cell material [4]. This article nicely expands upon existing technological obstacles and the lack of human donor material, a major limitation of their broader clinical use. The authors show that xenotransplantation might provide an ethically inoffensive and unlimited cell source for cardiac therapies. In this regard, the potential of porcine cells is extensively outlined in this article with a focus on cardiovascular regenerative medicine.

The final review article in this Special Issue by Lippi et al. addresses human cardiovascular disease modeling to obtain appropriate and reliable in vitro cell models in order to understand pathologic phenotypes and molecular mechanisms, as well as to develop therapies based on simple, reproducible techniques that are independent of animal experiments [5]. The authors summarize various cell types—including advantages and disadvantages—that are subjected to these goals, such as primary cells and embryonic stem cells with a particular focus on iPSCs as an advanced tool for personalized human disease modeling, diagnosis, and therapy. Importantly, Lippi et al. also introduced state-of-the-art multicellular and three-dimensional in vitro tissue constructs that reflect physiological in vivo conditions.

In their original article, Szepes et al. transferred this organoid approach to iPSC-derived pericyte-like cells that are useful for vascularization and fibrosis-related cardiac tissue remodeling in vitro [6]. They show that human pluripotent stem cells (hPSCs) bear the potential of de novo forming bioartificial cardiac tissue (BCT) using a variety of different cardiovascular cell types. This served to model the pathogenesis of myocardial interstitial fibrosis (MIF), which leads to excessive extracellular matrix deposition, increased myocardial stiffness, functional weakening and compensatory cardiomyocyte hypertrophy. As MIF is difficult to study in patients due to multiple pathomechanisms and the lack of available patient samples, the authors developed this BCT in vitro model of MIF to investigate the interaction of different cell types (hPSC-derived cardiomyocytes, endothelial cells, pericytes and primary fibroblasts). In these in vitro constructs, iPSC-pericytes improved sarcomere organization and supported vascularization. Moreover, EC- and pericyte-mediated effects on fibrosis-related cardiac tissue remodeling could be validated. The full benefit of such constructs will depend on the availability of truly mature in vitro-generated cardiomyocytes, which is still a critical point. In their original article, Lemcke et al. address this important topic by introducing a super-resolution microscopy-based approach for quantitatively evaluating the structural maturation of iPSC-derived cardiomyocytes [7]. In particular, a well-structured sarcomere apparatus is a prerequisite of functionally mature cardiomyocytes. Relying on the photoactivated localization microscopy (PALM) of α-actinin fluorescence-labelled cells, highly resolved images for measuring sarcomere length and z-disc thickness were achieved. It was an interesting key finding here that iPSC-derived and neonatal cardiomyocytes were highly similar in their (immature) sarcomere organization, yet the contraction capacity was even inferior in iPSC-derived cardiac cells, underlining the importance of further optimized in vitro differentiation protocols that can be monitored utilizing this novel method.

Human mesenchymal stromal cells (hmMSC) from healthy and dilated myocardium represent an interesting alternative cell source to generate cardiomyocytes for therapy as well as disease modeling. In their original article, Miksiunas et al. investigated the effects of the histone deacetylase (HDAC) inhibitor suberoylanilide hydroxamic acid (SAHA) on energy metabolism and the cardiac differentiation of these cells [8]. In explant outgrowths, cell proliferation, HDAC activity, mitochondrial membrane potential and ATP levels were determined, as well as Nkx2.5, cardiac troponin T and α-actinin expression and mitochondrial activity. From their results, the authors conclude that HDAC inhibition might become an important therapeutic tool to treat dilated cardiomyopathy (DCM) as human dilated myocardium-derived MSCs appear to retain regenerative potential, possibly further stimulated by HDAC inhibitors such as SAHA.

Inflammation is crucial for the healing process post-myocardial infarction and is therefore of great interest for therapeutic as well as prognostic purposes. Therefore, Vasudevan et al. developed the longitudinal imaging of myocardial inflammation after infarction in mice using 18F-FDG (18F-Fluorodeoxyglucose) positron emission tomography (PET), which is described in the final original article of this Special Issue [9]. However, for PET imaging, a technical hurdle existed: While monocytes and macrophages are metabolically highly active and accumulate the glucose analog 18F-FDG, for the specific allocation of the radioactivity to these inflammatory cells, glucose metabolism in viable myocardium needs to be shut off. The authors have solved this problem by introducing a strategy for systematic image analysis, a prerequisite to evaluate therapies targeting myocardial inflammation. This method, for the first time, enables the acquisition of comparable data in mouse infarction studies, setting the basis for the PET-based assessment of myocardial inflammation as a prognostic tool.

In summary, our Special Issue has gathered important reviews as well as original articles on state-of-the-art research in regenerative cardiovascular medicine. We are highly confident that these will be of significant interest for colleagues in the field.
